# Atypical ductal hyperplasia and the risk of underestimation: tissue sampling method, multifocality, and associated calcification significantly influence the diagnostic upgrade rate based on subsequent surgical specimens

**DOI:** 10.1007/s12282-018-00943-2

**Published:** 2018-12-27

**Authors:** Christoph J. Rageth, Ravit Rubenov, Cristian Bronz, Daniel Dietrich, Christoph Tausch, Ann-Katrin Rodewald, Zsuzsanna Varga

**Affiliations:** 1 Centre du sein, Département de Gynécologie et d’Obstétrique, Hôpitaux Universitaires de Genève, Bd de la Cluse 30, 1211 Geneva 14, Switzerland; 2 Brust-Zentrum Zürich, Seefeldstr. 214, 8008 Zurich, Switzerland; 3 Clinic for Gynecology, University Hospital Zurich, 8091 Zurich, Switzerland; 4 Swiss Group for Clinical Cancer Research, Bern, Switzerland; 5 Institute of Pathology and Molecular Pathology, University Hospital Zurich, Schmelzbergstrasse 12, 8091 Zurich, Switzerland; 6 Ringlikerstrasse 53, 8142 Uitikon Waldegg, Switzerland

**Keywords:** B3 lesion, Atypical ductal hyperplasia, ADH, Underestimation

## Abstract

**Background:**

Risk assessment and therapeutic options are challenges when counselling patients with an atypical ductal hyperplasia (ADH) to undergo either open surgery or follow-up only.

**Methods:**

We retrospectively analyzed a series of ADH lesions and assessed whether the morphological parameters of the biopsy materials indicated whether the patient should undergo surgery. A total of 207 breast biopsies [56 core needle biopsies (CNBs) and 151 vacuum-assisted biopsies (VABs)] histologically diagnosed as ADH were analyzed retrospectively, together with subsequently obtained surgical specimens. All histological slides were re-analyzed with regard to the presence/absence of ADH-associated calcification, other B3 lesions (lesion of uncertain malignant potential), extent of the lesion, and the presence of multifocality.

**Results:**

The overall underestimation rate for the whole cohort was 39% (57% for CNB, 33% for VAB). In the univariate analysis, the method of biopsy (CNB vs VAB, *p* = 0.002) and presence of multifocality in VAB specimens (*p* = 0.0176) were significant risk factors for the underestimation of the disease (ductal carcinoma in situ or invasive cancer detected on subsequent open biopsy). In the multivariate logistic regression model, the absence of calcification (*p* = 0.0252) and the presence of multifocality (unifocal vs multifocal ADH, *p* = 0.0147) in VAB specimens were significant risk factors for underestimation.

**Conclusions:**

Multifocal ADH without associated calcification diagnosed by CNB tends to have a higher upgrade rate. Because the upgrade rate was 16.5% even in the group with the lowest risk (VAB-diagnosed unifocal ADH with calcification), we could not identify a subgroup that would not require an open biopsy.

## Introduction

Atypical ductal hyperplasia (ADH) is a small, mostly unifocal, low-grade intraductal lesion in the breast, which in most cases is detected by the associated calcification seen on mammograms [1–4]. The histological criteria for ADH have been clearly defined as the involvement of no more than one terminal ductal lobular unit or low-grade intraductal proliferation with a maximum size of 2 mm, and a failure to meet all the criteria for low-grade ductal carcinoma in situ (DCIS) [1]. The definition of ADH is similar to that of low-grade DCIS, and the clinical management strategy has been surgery for over 2 decades, because the risk of underestimating the disease is 20–30%, calculated from a comparison of the diagnosis based on needle biopsy and the subsequent analysis of surgical specimens [2, 5, 6]. However, in recent years, there has been an increasing debate over whether all ADH lesions should be treated surgically or in select cases, should receive only follow-up after a radiographically identified lesion has been removed by vacuum-assisted biopsy (VAB) [7, 8]. The biological parameters (i.e., biomarkers) of ADH include only histological criteria as the lesions are too small to evaluate the prognostic values of various antibodies with tissue microarrays, because multiple sections of sufficient thickness cannot be obtained [9–13]. Therefore, the criteria for ADH that have been used to predict the clinical outcome after its diagnosis and to provide an algorithm for selecting appropriate patients for a wait-and-see management are predominantly histological parameters, including the exact size of the lesion and the presence of associated calcification, necrosis, and multifocality together with radiographic findings such as the presence of calcification or mass lesions [1, 4, 14]. Previous studies have differed in their design and case loads, resulting in a rather wide range of diagnostic underestimation and an emphasis on certain criteria to develop a reliable algorithm for selecting patients for conservative treatment [8, 15–20].

In our study, we collected histological slides from 207 patients diagnosed with ADH with either a preoperative diagnostic core needle biopsy (CNB) or VAB and correlated the histological findings with the biopsy method and the diagnostic outcome after a subsequent open biopsy. Our aim was to identify any histological parameters predictive of outcome in any subgroup of patients.

## Materials and methods

We analyzed the clinicopathological data of 207 patients histologically diagnosed with ADH, classified as B3 (lesion of uncertain malignant potential), or B4 (suspicious of malignancy), by CNB or VAB, who had undergone subsequent open surgery in 2002–2015. Of the 207 diagnostic biopsies, 56 were CNBs and 151 were VABs. All biopsies were performed at the Breast Center Seefeld Zurich and were histologically processed at the Institute of Pathology and Molecular Pathology of the University Hospital Zurich, Switzerland.

### VABs

In 2002–2015, 5213 VABs were performed at the Breast Center Seefeld Zurich, using Encore^®^ (Bard, Tempe, AZ, USA), Suros Eviva^®^ (Hologic^®^, Marlborough, MA, USA), or Mammotome^®^ (Devicor Medical Products, Cincinnati, OH, USA). In general, 7G or 9G needles were used for the biopsies performed under magnetic resonance imaging (MRI) and ultrasound (US), and 11G needles were used for stereotactic or tomosynthesis-guided biopsies. All procedures were performed under local anesthesia, except in two patients who required general anesthesia because multiple bilateral lesions were removed under US guidance.

Of the 5213 VABs, 1722 were guided by US (handheld), 125 by MRI, and 3366 by stereotactic surgery (after 2012, by tomosynthesis). The main indication for stereotactic or tomosynthesis-guided VAB was suspicious microcalcifications, and the indications of asymmetric density and architectural distortion were observed in only a few patients. MRI-guided VAB was indicated when a suspicious MRI finding could not be confirmed by mammogram or a second look US. The indication for handheld VAB was a US finding requiring histological clarification. As well as benign findings (71.30%), DCIS (10.5%), and invasive cancers (3.95%), 13.8% of the specimens showed cellular atypia, and 191 (3.7%) of these were classified as ADH.

### CNBs

In 2001–2015, 15,528 US-guided CNBs (with 14G needles) were performed, 66 of which indicated a diagnosis of ADH.

The decision to perform initially either CNB or VAB depended on the imaging characteristics. CNB was the preferred method in case of a visible, mostly mass lesion on ultrasound while VAB was the preferred method in cases with mammographically detected calcification not visible at ultrasound.

### Cases of ADH included in the study

We included in this study only patients with ADH who had undergone subsequent open surgery and for whom it was possible to correlate the ADH diagnosis based on VAB or CNB with that based on the surgical specimen.

### Statistical methods

The statistical analyses were performed with frequency tables and univariate and multivariate logistic regression models. All statistical analyses were performed by the Swiss Group for Clinical Cancer Research, Bern, Switzerland.

### Histological processing and findings

All biopsy materials were fixed in 4% neutral buffered formalin and embedded in paraffin blocks with routine histological processing. Sections (2 µm thick) were prepared from the paraffin blocks; different levels from each block were cut separately and stained routinely with hematoxylin and eosin.

### Histological criteria

For all biopsies, the diagnosis of ADH was made according to the current World Health Organization classification of breast tumors [21]. ADH was defined as a focal lesion of monotonous low-grade intraductal cell proliferation with secondary architectural formation of Roman bridges [1, 6, 22, 23]. An ADH was defined both quantitatively as one ADH focus (max. 2 ducts involvement and/or max. 2 mm in max. dimension and called as B3 category) and also qualitatively as multifocal (if in more than one biopsy cylinder an ADH focus was identified and /or > 2 ducts were involved in max. dimension of 2 mm each, and categorized as B4 category) [1, 6, 22, 23].

**Fig. 1:**
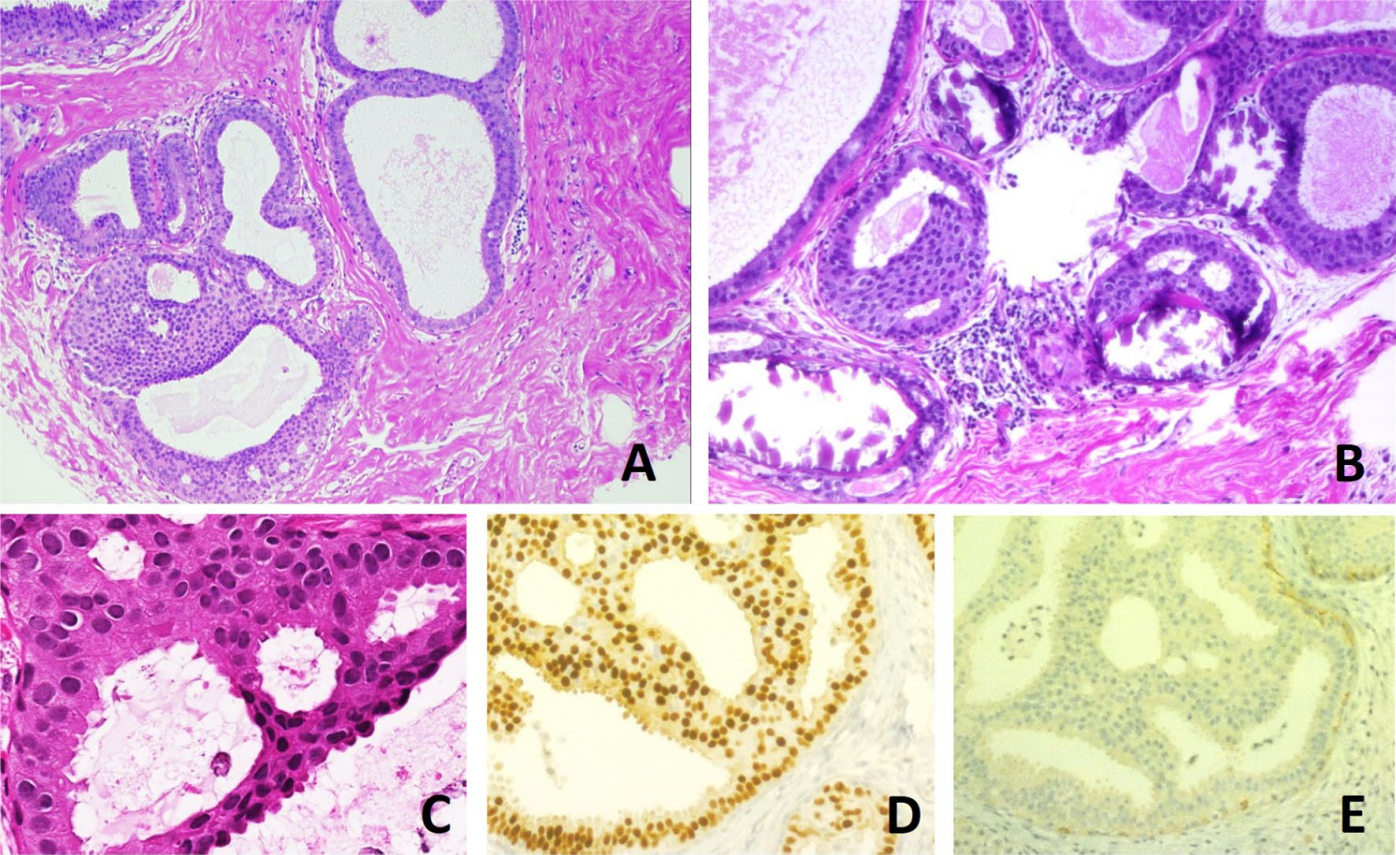
Histological appearance of atypical ductal hyperplasia (ADH) and immunohistochemical phenotype. **a** One focus (< 2 mm) of two architecturally disarranged cross sections of tubuli showing a monotonous intraductal proliferation with secondary intraluminal architecture. Hematoxylin and Eosin stain. **b** One area of an ADH with associated calcifications intraluminal. Hematoxylin and Eosin stain. **c** Higher magnification of ADH shows low-grade nuclear atypia and monotonous cell proliferation along with secondary intraluminal architecture. Hematoxylin and Eosin stain. **d** Strong and uniform expression of estrogen receptors (ER). ER immunohistochemistry. **e** Lack of basal cytokeratins (CK5/6). CK5/6 immunohistochemistry

The presence of calcification and multifocality (as B3 or B4 category) was noted. Findings additional to ADH, including lobular neoplasia (LN), radial scar (RS), flat epithelial atypia (FEA) and intraductal papilloma, were noted in the reports. In many cases, immunohistochemical analyses of estrogen receptors and basal cytokeratins 5/6 were also performed to confirm the ADH diagnosis. The diagnosis of ADH was confirmed if immunohistochemistry showed the diffuse upregulation of estrogen receptors and negativity for basal cytokeratins [21] (Fig. 1).

The whole cohort underwent a retrospective review, and the histological (including immunohistochemical) slides were reviewed by two pathologists who are specialists in breast pathology (AKR, ZV). The following features were assessed in the review: the histological diagnosis of ADH, the presence of multifocality in the biopsies, the presence of necrosis and associated calcification, and the extent of the lesion (in millimeters). The presence of FEA, RS, LN, and papilloma was also noted in the histological review.

Additionally, an age parameter (cut-off at 50 years) was added to the criteria.

‘Upgrade’ was defined as a diagnosis of DCIS or invasive carcinoma based on the subsequently obtained surgical specimens (Details are shown in Table 1).

**Table 1:** Outcome of subsequent open surgery stratified by histological parameters on core needle biopsy (CNB, 14G) and vacuum-assisted biopsy (VAB, 7–11G)

Histological and biopsy characteristics	CNB; *n* = 56		VAB; *n* = 151		
	Underestimation				
	No	Yes		No	Yes
Method of biopsy (CNB vs VAB)	24 (42.86%)	32 (57.14%)		101 (66.89%)	50 (33.11%)
ADH					
Pure	18 (40.91%)	26 (59.09%)		39 (62.90%)	23 (37.10%)
Not pure	6 (50.00%)	6 (50.00%)		62 (69.66%)	27 (30.34%)
FEA					
Absent	17 (40.48%)	25 (59.52%)		42 (68.85%)	19 (31.15%)
Present	7 (50.00%)	7 (50.00%)		59 (65.56%)	31 (34.44%)
RS					
Absent	24 (42.86%)	32 (57.14%)		94 (65.73%)	49 (34.27%)
Present	–	–		7 (87.50%)	1 (12.50%)
LN					
Absent	24 (42.86%)	32 (57.14%)		90 (67.16%)	44 (32.84%)
Present	–	–		11 (64.71%)	6 (35.29%)
Calcifications					
Absent	19 (45.24%)	23 (54.76%)		19 (55.88)	15 (44.12%)
Present	5 (35.71%)	9 (64.29%)		82 (70.09%)	35 (29.91%)
Diameter					
1.1–2 mm	9 (40.91%)	13 (59.09%)		49 (69.01%)	22 (30.99%)
Up to 1 mm	15 (44.12%)	19 (55.88%)		52 (65.00%)	28 (35%)
Multifocality					
Multifocal	4 (36.36%)	7 (63.64%)		34 (55.74%)	27 (44.26%)
Unifocal	20 (44.44%)	25 (55.56%)		67 (74.44%)	23 (24.56%)
Papilloma					
Absent	21 (42.86%)	28 (57.14%)		93 (65.49%)	49 (34.51%)
Present	3 (42.86%)	4 (57.14%)		8 (88.89%)	1 (11.11%)

Underestimation is “yes”, when DCIS or invasive cancer was found on subsequent open surgery

*ADH* atypical ductal hyperplasia, *FEA* flat epithelial atypia, *RS* radial scar, *LN* lobular neoplasia, classical type. Pure ADH: absence of FEA, RS, LN or papilloma in the biopsy

## Results

Of the patients with ADH, 207 biopsies (151 VABs and 56 CNBs) could be re-analyzed by reviewing the histological slides and classifying ADH according to the histological criteria mentioned above.

The histological findings of additional B3 lesions are listed in Table 2. In the univariate logistic regression analysis of disease underestimation, the biopsy method (CNB vs VAB, *p* = 0.002) and the presence of multifocality in the VAB specimens (multifocal vs unifocal ADH, *p* = 0.0176) were significant risk factors (Tables 3, 4). The multivariate logistic regression analysis of disease underestimation also identified the absence of calcification (*p* = 0.0252) and the presence of multifocality (unifocal vs multifocal ADH, *p* = 0.0147) in VAB specimens as significant risk factors. The other parameters including the age parameter showed no significant association with the rate of disease upgrade based on subsequent surgical specimens. We did not identify large comedo type necrosis, however, smaller necrotic foci in association with calcification were observed. In the surrounding breast tissue, no relevant calcifications were found.

**Table 2:** Histological factors and additional B3 lesions, according to the biopsy method

Histological and biopsy characteristics	Core needle biopsy (CNB); *n* = 56	Vacuum-assisted biopsy (VAB); *n* = 151
ADH		
Pure	44 (78.57%)	62 (41.06%)
Not pure	12 (21.93%)	89 (58.94%)
FEA		
Absent	42 (75.00%)	61 (40.40%)
Present	14 (25.00%)	90 (59.60%)
RS		
Absent	56 (100%)	143 (94.70%)
Present	0 (0%)	8 (5.30%)
LN		
Absent	56 (100%)	134 (88.74%)
Present	0 (0%)	17 (11.26%)
Calcifications		
Absent	42 (75.00%)	34 (22.52%)
Present	14 (25.00%)	117 (77.48%)
Diameter of ADH		
1.1–2 mm	22 (39.29%)	71 (47.02%)
Up to 1 mm	34 (60.71%)	80 (52.98%)
Multifocality		
Multifocal	11 (19.64%)	61 (40.40%)
Unifocal	45 (80.36%)	90 (59.60%)
Papilloma		
Absent	49 (87.50%)	142 (94.04%)
Present	7 (12.50%)	9 (5.96%)

*ADH* atypical ductal hyperplasia, *FEA* flat epithelial atypia, *RS* radial scar, *LN* lobular neoplasia, classical type. Pure ADH: absence of FEA, RS, LN or papilloma in the biopsy

**Table 3:** Univariate and multivariate logistic regression analyses of disease underestimation based on core needle biopsy (CNB) (*N* = 56)

Effect	Univariate				Multivariate			
	Odds ratio	95% Wald confidence limits		*p* value	Odds ratio	95% Wald confidence limits		*p* value
ADH	1.44	0.40	5.20	0.574	1.51	0.14	16.89	0.738
FEA	0.68	0.20	2.29	0.534	0.68	0.13	3.60	0.651
RS	–				–			
LN	–				–			
Extension	1.14	0.39	3.38	0.813	0.94	0.30	3.03	0.923
Multifocality	1.40	0.36	5.47	0.628	1.48	0.35	6.26	0.596
Calcification	1.49	0.43	5.19	0.534	1.76	0.41	7.46	0.445
Papillomas	1.00	0.20	4.96	0.999	1.88	0.15	23.44	0.625

Associated B3 lesions, diameter of the lesion, multifocality, or calcification had no significant effect on upgrade rates in core needle biopsy samples (14G)

*ADH* atypical ductal hyperplasia, *FEA* flat epithelial atypia, *RS* radial scar, *LN* lobular neoplasia, classical type

**Table 4:** Logistic regression models for underestimation in vacuum-assisted biopsies (VAB) (*N* = 151)

Effect	Univariate				Multivariate			
	Odds ratio	95% Wald confidence limits		*p* value	Odds ratio	95% Wald confidence limits		*p* value
ADH	1.35	0.68	2.69	0.386	1.22	0.40	3.67	0.730
FEA	1.16	0.58	2.33	0.673	1.35	0.45	4.05	0.592
RS	0.27	0.03	2.29	0.232	0.29	0.03	2.97	0.295
LN	1.12	0.39	3.21	0.839	0.84	0.25	2.83	0.783
Extension	0.83	0.42	1.65	0.601	0.85	0.41	1.77	0.662
Multifocality	2.31	1.16	4.62	0.018*	2.66	1.22	5.80	0.014*
Calcification	0.54	0.25	1.18	0.124	0.35	0.14	0.88	0.025*
Papillomas	0.24	0.03	1.95	0.181	0.33	0.03	3.31	0.346

Univariate and multivariate logistic regression analyses of disease underestimation by vacuum-assisted biopsy. Associated B3 lesions or the diameter of the lesion had no significant effect, but multifocality and associated calcification significantly affected the upgrade rates for vacuum-assisted biopsy samples (7–11G)

*ADH* atypical ductal hyperplasia, *FEA* flat epithelial atypia, *RS* radial scar, *LN* lobular neoplasia, classical type

*Statistically significant

The upgrade rate in the cohort with the lowest risk (unifocal ADH, calcification present, and VAB method) was 16.5% (10 DCIS and 1 invasive cancer in 63 patients). The overall upgrade rate in the whole cohort was 39% (57% by CNB, 33% by VAB).

## Discussion

To the best of our knowledge, this is the largest study to show that the biopsy method (CNB or VAB) influences the outcome determined based on surgical specimens, with a greater rate of disease upgrade after diagnosis by CNB than by VAB. In one previous study, Badan et al. reported similar observations in 40 patients [24]. Our case series of 207 patients confirms their observation. Although it has been reported that residual calcification and mass lesions or opacity on mammograms should be considered risk factors for upgrading a diagnosis of ADH, the biopsy method used for diagnosis must also be considered [1, 8, 14, 20, 25, 26]. The importance of the biopsy method should be integrated into clinical guidelines for B3 lesions, as in the First International Consensus Conference on B3 lesions, which made distinct therapeutic recommendations depending on the method of biopsy [8].

Several previous studies have analyzed the relationships between pathological parameters (including extent of ADH, degree of atypia, and presence of necrosis) with the outcome determined from surgical specimens [1, 15, 17, 19, 27]. These histological biomarkers tend to correlate with the rate of diagnostic upgrade, but none of them alone reliably predicts the need for open surgery rather than conservative management in individual cases. Patients with necrosis and multifocality (at least three ducts or more than one biopsy core involved) reportedly have a significantly higher diagnostic upgrade rate than patients lacking these factors, suggesting that these patients should undergo open surgery [7, 13, 20, 25, 26]. However, in those studies, radiographic findings were also considered in making the therapeutic recommendation. In particular, the presence of mass lesions, opacity, and residual calcification after VAB were factors favoring surgical intervention. Regarding calcification within the ADH lesion, Khoury et al. [28] found the opposite to our finding: if calcification was associated with ADH, the risk for upgrade was 1.9 times higher, while it was by a factor of 0.68 lower in our analysis.

Our results partly support those of previous studies regarding the importance of calcification. Our study shows that calcification predicts diagnostic upgrade only in cases of ADH diagnosed by VAB, with a significantly lower upgrade rate in patients with calcification. The upgrade rate in cases of ADH diagnosed by CNB was independent of the presence of calcification. A relationship between calcification and diagnostic upgrade has been reported in previous studies [29, 30]. Our data provide further evidence that ADH without associated calcification requires further diagnostic steps, especially in patients diagnosed by CNB.

It can be assumed, that ADH cases detected on VAB lacking calcifications were most likely representing a non calcified extension of DCIS, which was not detectable as such in mammography.

The data reported in the literature on multifocality and the diagnostic upgrade of ADH are controversial [19, 20, 25]. As well as multifocality, factors such as larger size (just around 2 mm), more extensive ADH lesions, and B4 category lesions (suggestive of, but not diagnostic for DCIS) have been mentioned, which makes any interpretation of the data difficult [17, 19, 20, 25]. We restricted our ADH cases to B3 lesions and including also multifocal lesions (B4 category). We have shown that if only one terminal ductal lobular unit is involved, the upgrade rate is significantly lower after VAB, but not after CNB.

Interestingly, as in our data, the data in the literature show fairly consistently that there is no association between ADH upgrade and the presence of other B3 lesions, such as LN, papilloma, RS, or FEA [4, 5, 8, 16, 17, 23, 31]. We could not confirm an association between additional B3 lesions and the diagnostic upgrade rate.

One earlier study by Hartman et al. reported a significant interaction between premenopausal status and histological findings. Breast cancer risk among patients < 45 years at the time of ADH diagnosis, was 6.99 times the expected risk, which was significantly lower (3.37) in postmenopausal patients (> 55 years), as was shown in Fig. 2 in this paper [32]. Interestingly, this interaction could not be confirmed in our study, probably due to the fact, that there were less premenopausal patients in our cohort.

In conclusion, we have provided evidence that the biopsy method used (CNB or VAB) influences the diagnostic upgrade rate. Especially in cases of ADH diagnosed by VAB, the presence of calcification in a unifocal lesion is associated with a significantly lower disease upgrade rate than is the absence of calcification and/or the presence of multifocality. These factors should be considered, in addition to imaging characteristics, when deciding whether a patient should undergo open surgery or simply be managed conservatively. These data require validation in further studies.
